# Autonomic maturation from birth to 2 years: normative values

**DOI:** 10.1016/j.heliyon.2019.e01300

**Published:** 2019-03-07

**Authors:** Hugues Patural, Vincent Pichot, Sophie Flori, Antoine Giraud, Patricia Franco, Patrick Pladys, Alain Beuchée, Frédéric Roche, Jean-Claude Barthelemy

**Affiliations:** a Neonatal Intensive Care Unit, Pediatric Department, University Hospital of Saint-Etienne, France; b EA SNA-EPIS 4607 Research Laboratory, University Saint-Etienne, France; c Pediatric Sleep Unit, Mother-Child's Hospital & INSERM U1028, University Lyon 1, France; d Signal and Image Processing Research Laboratory, INSERM-U1099, University Rennes 1, France

**Keywords:** Neuroscience

## Abstract

**Background:**

While heart rate variability (HRV) constitutes a relevant non-invasive tool to assess the autonomic nervous system (ANS) function with recognized diagnostic or therapeutic implications, there is still a lack of established data on maturation of autonomic control of heart rate during the first months of life. The Autonomic Baby Evaluation (*AuBE*) cohort was built to establish, the normal autonomic maturation profile from birth up to 2 years, in a healthy population of full-term newborns.

**Methods:**

Heart rate variability analysis was carried out in 271 full-term newborns (mean gestational age 39 wGA + 5 days) from reliable polysomnographic recordings at 0 (n = 270) and 6 (n = 221) months and from a 24-hour ambulatory electrocardiogram (ECG) at 12 (n = 210), 18 (n = 197), and 24 (n = 190) months. Indices of HRV analysis were calculated through the *ANSLabTools* software.

**Results:**

Indices are dissociated according a temporal, geometrical, frequency, Poincaré, empirical mode decomposition, fractal, Chaos and DC/AC and entropy analysis. Each index is presented for five different periods of time, 0, 6, 12, 18 and 24 months and with smoothed values in the 3rd, 10th, 50th, 90th and 97th percentiles. Data are also presented for the full cohort and individualized by sex to account for gender variability.

**Discussion & conclusion:**

The physiological autonomic maturation profile from birth to 2 years in a healthy population of term neonates results in a fine-tuning autonomic maturation underlying progressively a new equilibrium and privileging the parasympathetic activity over the sympathetic activity.

## 1 Introduction

Whatever the age [1, 2], heart rate variability (HRV) referring to beat-to-beat variations of the RR interval length constitutes a relevant non-invasive tool to assess the autonomic nervous system (ANS) function with recognized diagnostic or therapeutic implications [3, 4, 5, 6, 7, 8, 9].

For the neonatologist, the interest in HRV lies in the perception of the status of congenital or acquired autonomic dysregulation, particularly the cardiorespiratory control as objective risk factor of Sudden infant death syndrome (SIDS) [10, 11, 12, 13, 14]. In the triple risk model of SIDS involving “vulnerable child, exogenous stress and critical developmental period”, the cardiorespiratory autonomic immaturity and abnormal awakening responses appear to be critical [11, 14]. The cardiorespiratory modulations observed during arousal are mediated by the cortico-hypothalamic pathways and cardiorespiratory nuclei of the brainstem, including the solitary tract, ambiguous and dorsal nucleus of the pneumogastric nerve. Kato et al. provided an epidemiological link between central abnormalities of cardiorespiratory response during awakening and sudden death, in a large survey including 20,000 children [15]. The links with molecular research analyzes contributions of inhibitors neurotransmitters of cardiorespiratory control, in the genesis of both apnea and bradycardia as GABA γ aminobutyric acid, adenosine, serotonin, endorphins and prostaglandins [16, 17] with the identification of an abnormal serotonergic response in the bulbar and arcuate nucleus of the hypothalamus in as much as 50% of the cases, presumably linked to genetic polymorphisms [18, 19, 20, 21]. In that view, overexpression of cardiac muscarinic receptors as well as an increased enzymatic acetylcholinesterase activity have been reported [22]. Regardless of the fetal risk associated with autonomic dysfunction, the study of the child autonomic status is also of great interest in many clinical situations as frequent as infections, sepsis [23, 24], chronic inflammatory [25] diseases or type 1 diabetes [26] in which impaired autonomic control and increased risk for cardiovascular disease are reported.

While there are advances on the biological regulations, there is still a lack of established data on maturation of autonomic, particularly during the first months of life. It has been established that at least 37 weeks of in utero maturation are needed to achieve full autonomic maturation at birth [2, 27, 28, 29, 30]. As a matter of fact, both cardiac autonomic responsiveness and baroreflex loop are altered in preterm infants regardless of gestational age (GA) and remain very low at theoretical term when compared with full-term newborns [27, 28]. In a recent review about ANS in newborns, an increasing postnatal age is related to a significant rise of HRV parameters, particularly for the high frequency (HF) parameter, recognized as a relevant representative of parasympathetic maturation [31].

While an autonomous imbalance in the first year of life implies inappropriate cardiorespiratory reactions to internal or environmental stress [11, 12, 13, 14], the lack of references data at that age makes clinical studies unrelated to the others. This may concern up to the premature adolescent, but focusing up the two first years of age may help monitoring the most at risk infants [32, 33, 34].

Therefore, describing the natural evolution of autonomous maturation in the first years of life may bring useful data to clinicians in search for a better interpretation of autonomic status and its clinical relationships. The Autonomic Baby Evaluation (*AuBE*) cohort was built to establish, for the first time, the normal autonomic maturation profile from birth. We here publish the first results of the cohort, up to 2 years, in that healthy population of full-term newborns [35].

## 2 Methods

### 2.1 Design

Autonomic Baby Evaluation (*AuBE*) study is a prospective single-center observational study conducted at the University Hospital of Saint-Etienne (France), a level III Maternity managing 3.500 births annually. The cohort of the consecutive full-term newborns was performed over a 24 months' period, from September 2009 to September 2011 to assess the autonomic maturation profile during the first two years of life.

For the Heart rate variability analysis, polysomnographic recordings were realized at 0 and 6 months and due to the growing motor ability of children, the recordings were then performed through a 24-hour ambulatory electrocardiogram (ECG) at 12, 18, and 24 months. The first two-year follow-up of the cohort ended in September 2014. All the design of the study with the approval by our institute's ethics committee, the inclusion and exclusion criteria and the technical procedure for acquiring recorded data have been published in a previous review [35]. The study was registered in the International Clinical Trials Registry under the label ClinicalTrials.gov ID NCT01583335 [29].

Among the 302 children recruited, we kept 271 (89.7%) of them as being full-term, excluding the 31 (10.3%) preterm born before 37 weeks of gestational age (wGA). They were recruited continuously according to the availability of the recorders independently of ethnicity, and sex. For term newborns (n = 271), the median gestational age was 39 wGA + 5 days [37 wGA, 41 wGA + 6 d]. The ratio M/F was 1.22 (149/122). We did not retain alcohol exposure during fetal life, but 86 (31%) newborns were probably exposed to nicotine in utero without precision of intensity [35].

No child presented a dysmorphic syndrome. At birth the mean term newborn weight was 3256 g (2640–4410), the mean length was 49.6 cm (44–55), and the mean head circumference was 34.2 cm (33.0–38.5). The mean Apgar score was 9 at one, 10 at two, and 10 at ten minutes. The mean CRIB score [36] was 0. One child received a brief resuscitation in the delivery room requiring a short cardiac massage with immediate hemodynamic recovery. No child presented neonatal lung disease, or hemodynamics, gastrointestinal or neurological troubles. One child (0.2%) had been treated for a suspected neonatal infection. Hospital stays lasted an average of 3 days [2, 5] and 100% of the children were considered clinically normal. No home monitoring was recommended at discharge. In this cohort of 271 infants, one child (0.03%) died in the third month of life, probably because of asphyxiation in bed without further explanation despite an extensive research of etiologic factors and an autopsic procedure. As a result, he was not excluded from the first recording. We note an early loss to follow-up before two years of age for 68/271 (25%) infants. Reliable HRV recordings were 270 polysomnographic recordings (M0) 210 polysomnographic recordings and 11 Holter 24 hours ECG (M6) and 210, 197 and 190 Holter ECG recordings at, respectively M12, M18 and M24.

### 2.2 Materials

The 24-hour ECG recordings were obtained from the polysomnographic recordings (*Dream® Medatec, Belgium*), or from ECG Holter *(Vista®, Novacor, Rueil Malmaison, France)*. The sampling frequency was 200 Hz for both materials. HRV calculations were performed through the *ANSLabTools* software [37].

First, ECG tracings were resampled at a frequency of 1000 Hz and each R peak was detected to provide the R-R interval series with a precision of 1 ms. Then, missing beats, isolated premature heart beats and artifacts were corrected using a spline cubic interpolation [38, 39].

### 2.3 Analysis of heart rate variability

Indices of HRV analysis were calculated through the *ANSLabTools* giving several types of indices according to the litterature [37, 38].

- **Time domain analysis**. They are based on means and standard deviations calculated on short-term to long-term variations in RR intervals. The standard deviation of normal-to-normal intervals (SDNN), the standard deviation of the mean of all normal RR intervals for 5-min segments (SDANN) and the mean of the standard deviation of all normal RR intervals for all 5-min segments (SDNNIDX) represents global and long-term variations [37, 38], and the percent difference between adjacent normal RR intervals greater than 50 ms (pNN50), the square root of the mean of the sum of the squared differences between adjacent normal RR intervals (rMSSD) represents fast changes linked to the parasympathetic activity. The geometrical indices calculated on the sample density distribution of the RR intervals, corresponds to the assignment of the number of equally long RR intervals to each value of their length.
Poincaré plot is a graphic tool using SD1 and SD2 index, calculated as the standard deviation of the distances of the RR intervals from they = x line and the y = −x + 2 mean (RR) line respectively. SD1 represents short-term and SD2 long-term RR intervals variability.
- **Frequency domain analysis**. For those analyses, the RR signal was resampled at 4 Hz and the high frequency (HF) bandwidth was extended up to 2 Hz as recommended for newborns and children analyses [37, 38, 39]. Whereas total power (Ptot) represents the global variability, ultra-low frequencies (ULF), very low frequencies (VLF), low frequencies (LF), and high frequencies (HF) represent specific bands of interest. HF power is modulated by parasympathetic modulation, LF power is controlled by both sympathetic and parasympathetic activity, and normalized indexes (LFnu, HFnu) or the LF/HF ratio (calculated has the mean of the LF/HF values of all successive 5-min epochs of the entire RR serie) are used to estimate sympathetic modulation and autonomic equilibrium.
- **Geometrical analysis.** The indices calculated are HRV triangular index (the integral of the density distribution divided by the maximum of the density distribution) and the TINN index (triangular interpolation of the RR interval histogram i.e. the baseline width of this triangle). These measures quantify overall HRV mainly influenced by slow, but not by rapid, oscillations of RR intervals.
- **Non-linear analysis**. The indices arising from nonlinear approach provide information on the complexity of the autonomic regulations. In this context, the fractality of heart rate variability consist in quantifying the repetition of patterns display at different scales. These indices were calculated using *detrend fluctuation analysis* (α_1_, α_2_, H), Hurst exponent, 1/f slope, Higuchi and Katz and largest Lyapunov exponent algorithms. In addition, entropy indices are an estimation of the regularity and complexity of pattern at different length. Many indices have been calculated as the Shanon entropy and its derived indices, conditional entropy, corrected conditional entropy, normalized corrected conditional entropy, the sample entropy and approximate entropy.
- **Deceleration and Acceleration Capacities.** In this last non-linear approach, these two indices are used to estimate the vagal and sympathetic capacities by analyzing heart deceleration capacity (DC: detection of sequences of two successive RR beats that increase) and acceleration capacity (AC: detection of all sequences that decrease).

### 2.4 Statistical analysis

A preliminary analysis showed that many HRV indices did not follow a Gaussian distribution even when log-transformed. Thus, to construct the centiles for 24h HRV indices from 0 to 24 months, we first search for the distribution that better fits the data. For each index, we evaluated the goodness of fit of various distribution using maximum likelihood estimates of the parameters and by visual inspection of the quantile-quantile plots of sample data versus theoretical quantiles of the distribution. The analysis showed that the best fit was made by a generalized extreme value distribution. Such a distribution is often used to model the smallest or largest value among a large set of independent, identically distributed random values representing measurements or observations. It combines three simpler distributions into a single form, allowing a continuous range of possible shapes that include all three of the simpler distributions. The three distribution types correspond to the limiting distribution of block maxima from different classes of underlying distributions: distributions whose tails decrease exponentially, such as the normal distribution; distributions whose tails decrease as a polynomial, such as Student's t distribution; distributions whose tails are finite, such as the beta distribution.

From this chosen distribution, we extracted the median and the centiles (3rd, 10th, 90th, 97th) for each HRV index at each period (0, 6^th^, 12^th^, 18^th^ and 24^th^ months). Then, the curves were plotted using polynomial curve fitting technic. Values were calculated for all children and separately for girls and boys.

HRV differences between girls and boys were calculated using a Mann-Whitney U test. The Friedman test was utilized to compare the HRV indices measured at each period (0, 6^th^, 12^th^, 18^th^ and 24^th^ months), for all subject and separately for girls and boys. A p-value was considered as statistically significant when <0,05.

Statistics and graphs were computed using Statistics and Machine Learning Matlab toolbox 10.2 R2016a (The MathWorks Inc., Natick, MA, USA).

## 3 Results

The results of the HRV indices are presented according to the fields previously described as temporal (Table 1), geometrical (Table 2), frequency (Table 3), Poincaré (Table 4), empirical mode decomposition (Table 5), fractal (Table 6), Chaos and DC/AC (Table 7), and entropy analysis (Table 8). Each index is presented for 5 different periods of time, 0, 6, 12, 18 and 24 months and with smoothed values in the 3rd, 10th, 50th, 90th and 97th percentiles. Data are also presented for the full cohort (left) and individualized by sex to account for gender variability (center and right).

**Table 1:** HRV indices in the temporal domain (all 5-min segments).

			**Smoothed centiles for Mean RR (ms) (All)**							**Smoothed centiles for Mean RR (ms) (Girls)**							**Smoothed centiles for Mean RR (ms) (Boys)**			
Months	n						Months	n						Months	n					
		3rd	10th	50th	90th	97th			3rd	10th	50th	90th	97th			3rd	10th	50th	90th	97th
0	260	420	432	466	514	542	0	117	420	430	460	506	536	0	143	421	435	472	520	546
6	205	425	442	483	533	558	6	95	423	439	477	525	551	6	110	428	445	488	537	560
12	198	434	455	505	561	586	12	86	433	452	500	554	580	12	112	436	459	511	564	586
18	187	447	473	534	599	628	18	82	448	472	528	593	624	18	105	447	475	538	601	626
24	182	464	495	567	646	682	24	80	470	496	561	642	683	24	102	460	495	572	647	678
Total	1032						Total	460						Total	572					
			**Smoothed centiles for Mean HR (bpm) (All)**							**Smoothed centiles for Mean HR (bpm) (Girls)**							**Smoothed centiles for Mean HR (bpm) (Boys)**			
Months	n						Months	n						Months	n					
		3rd	10th	50th	90th	97th			3rd	10th	50th	90th	97th			3rd	10th	50th	90th	97th
0	260	111.3	116.7	128.4	139.3	143.5	0	117	112.5	118.3	130.2	140.2	143.6	0	143	110.6	115.6	127.0	138.3	142.9
6	205	108.3	112.9	124.0	136.3	142.0	6	95	109.3	114.2	125.5	137.5	142.9	6	110	107.8	112.1	122.7	135.0	141.0
12	198	103.5	107.8	118.7	132.3	139.3	12	86	104.2	108.7	120.0	133.4	140.0	12	112	103.2	107.2	117.6	131.1	138.4
18	187	96.9	101.2	112.4	127.3	135.5	18	82	97.0	101.8	113.7	128.0	135.1	18	105	97.0	100.9	111.5	126.5	135.3
24	182	88.5	93.2	105.3	121.5	130.6	24	80	87.9	93.4	106.5	121.2	128.1	24	102	89.1	93.2	104.5	121.2	131.6
Total	1032						Total	460						Total	572					
			**Smoothed centiles for pcNN20 (%) (All)**							**Smoothed centiles for pcNN20 (%) (Girls)**							**Smoothed centiles for pcNN20 (%) (Boys)**			
Months	n						Months	n						Months	n					
		3rd	10th	50th	90th	97th			3rd	10th	50th	90th	97th			3rd	10th	50th	90th	97th
0	260	2.84	6.50	17.21	34.53	46.20	0	117	2.33	5.35	14.93	32.75	46.40	0	143	4.03	7.92	18.92	35.88	46.87
6	205	5.70	10.38	22.69	39.30	48.78	6	95	4.41	8.93	20.77	37.13	46.97	6	110	7.38	11.95	24.16	40.83	50.28
12	198	7.78	13.81	28.45	45.46	53.70	12	86	6.21	12.26	26.77	43.32	51.23	12	112	9.33	15.33	29.91	46.83	55.01
18	187	9.11	16.81	34.56	53.09	61.03	18	82	7.73	15.37	32.99	51.41	59.21	18	105	9.89	18.04	36.12	53.85	61.04
24	182	9.67	19.35	40.94	62.11	70.67	24	80	8.98	18.23	39.37	61.29	70.84	24	102	9.07	20.11	42.83	61.92	68.38
Total	1032						Total	460						Total	572					
			**Smoothed centiles for pcNN30 (%) (All)**							**Smoothed centiles for pcNN30 (%) (Girls)**							**Smoothed centiles for pcNN30 (%) (Boys)**			
Months	n						Months	n						Months	n					
		3rd	10th	50th	90th	97th			3rd	10th	50th	90th	97th			3rd	10th	50th	90th	97th
0	260	1.27	2.90	8.88	23.45	37.84	0	117	1.32	2.38	7.12	22.57	41.97	0	143	1.57	3.60	10.29	24.25	36.40
6	205	1.91	4.25	11.64	25.95	37.79	6	95	1.54	3.54	10.12	24.43	38.16	6	110	2.62	5.08	12.79	27.17	38.40
12	198	2.36	5.78	15.45	30.73	41.11	12	86	1.81	4.95	14.03	29.00	39.68	12	112	3.11	6.64	16.57	32.01	42.33
18	187	2.63	7.49	20.34	37.84	47.85	18	82	2.12	6.64	18.90	36.33	46.53	18	105	3.04	8.27	21.59	38.72	48.15
24	182	2.70	9.38	26.27	47.19	57.94	24	80	2.47	8.58	24.66	46.34	58.65	24	102	2.42	9.98	27.87	47.33	55.89
Total	1032						Total	460						Total	572					
			**Smoothed centiles for pcNN50 (%) (All)**							**Smoothed centiles for pcNN50 (%) (Girls)**							**Smoothed centiles for pcNN50 (%) (Boys)**			
Months	n						Months	n						Months	n					
		3rd	10th	50th	90th	97th			3rd	10th	50th	90th	97th			3rd	10th	50th	90th	97th
0	260	0.56	1.11	3.62	13.08	27.49	0	117	0.51	0.92	2.92	11.53	26.57	0	143	0.64	1.37	4.37	13.75	25.98
6	205	0.52	1.12	3.80	13.46	27.53	6	95	0.47	0.92	3.09	12.12	26.75	6	110	0.63	1.37	4.44	14.33	27.54
12	198	0.50	1.42	5.12	16.48	31.07	12	86	0.47	1.20	4.36	15.32	30.77	12	112	0.59	1.66	5.76	17.40	31.58
18	187	0.49	2.01	7.61	22.17	38.16	18	82	0.51	1.77	6.73	21.16	38.67	18	105	0.52	2.24	8.33	22.95	38.05
24	182	0.50	2.88	11.24	30.47	48.72	24	80	0.59	2.63	10.19	29.59	50.37	24	102	0.41	3.12	12.15	30.96	46.97
Total	1032						Total	460						Total	572					
			**Smoothed centiles for rMSSD (ms) (All)**							**Smoothed centiles for rMSSD (ms) (Girls)**							**Smoothed centiles for rMSSD (ms) (Boys)**			
Months	n						Months	n						Months	n					
		3rd	10th	50th	90th	97th			3rd	10th	50th	90th	97th			3rd	10th	50th	90th	97th
0	260	13.5	16.6	26.8	47.5	65.1	0	117	13.2	15.8	24.6	43.2	59.7	0	143	14.1	17.6	28.8	50.4	68.3
6	205	13.0	15.9	25.8	48.2	69.6	6	95	12.4	15.0	24.0	44.1	62.9	6	110	14.0	16.9	27.2	51.4	75.4
12	198	13.1	16.3	27.9	55.0	81.5	12	86	12.3	15.4	26.2	50.7	74.3	12	112	14.0	17.3	29.2	58.3	88.2
18	187	13.6	18.0	33.1	67.7	100.8	18	82	13.1	17.1	31.2	63.3	94.0	18	105	14.3	18.8	34.6	71.2	106.4
24	182	14.6	20.7	41.4	86.4	127.3	24	80	14.5	20.0	38.9	81.5	121.8	24	102	14.7	21.4	43.5	89.9	130.3
Total	1032						Total	460						Total	572					
			**Smoothed centiles for SDANN (ms) (All)**							**Smoothed centiles for SDANN (ms) (Girls)**							**Smoothed centiles for SDANN (ms) (Boys)**			
Months	n						Months	n						Months	n					
		3rd	10th	50th	90th	97th			3rd	10th	50th	90th	97th			3rd	10th	50th	90th	97th
0	260	25.5	31.8	47.5	66.4	75.9	0	117	24.0	30.3	46.2	65.7	75.7	0	143	26.3	33.1	49.1	66.4	74.1
6	205	32.1	39.5	57.6	79.5	90.4	6	95	32.6	38.9	55.5	77.1	88.9	6	110	32.1	40.2	59.6	80.7	90.3
12	198	35.5	44.1	65.3	90.1	102.2	12	86	36.6	43.9	62.7	86.6	99.3	12	112	35.2	44.8	67.5	92.3	103.5
18	187	35.7	45.9	70.6	98.5	111.6	18	82	36.0	45.3	67.8	94.2	107.2	18	105	35.6	46.8	73.0	101.0	113.5
24	182	32.7	44.8	73.4	104.5	118.5	24	80	30.9	43.1	70.9	99.8	112.4	24	102	33.5	46.3	76.0	107.0	120.4
Total	1032						Total	460						Total	572					
			**Smoothed centiles for SDNNIDX (ms) (All)**							**Smoothed centiles for SDNNIDX (ms) (Girls)**							**Smoothed centiles for SDNNIDX (ms) (Boys)**			
Months	n						Months	n						Months	n					
		3rd	10th	50th	90th	97th			3rd	10th	50th	90th	97th			3rd	10th	50th	90th	97th
0	260	24.5	29.2	42.0	60.7	72.1	0	117	24.1	27.9	39.1	57.6	70.4	0	143	25.8	30.9	44.4	62.8	73.4
6	205	23.3	27.1	37.8	54.9	66.4	6	95	22.8	26.1	35.8	51.9	63.2	6	110	24.5	28.3	39.3	56.8	68.7
12	198	22.9	26.6	37.6	56.5	70.1	12	86	22.5	25.9	36.1	53.5	66.3	12	112	23.7	27.4	38.8	58.5	72.8
18	187	23.2	27.8	41.5	65.6	83.3	18	82	23.3	27.4	40.0	62.6	79.6	18	105	23.4	28.2	42.8	67.7	85.6
24	182	24.3	30.6	49.6	82.2	105.7	24	80	25.1	30.5	47.6	79.0	103.2	24	102	23.6	30.7	51.3	84.5	107.0
Total	1032						Total	460						Total	572					

HR (heart rate), SDNN (standard deviation of normal-to-normal intervals), SDNNIDX (mean of the standard deviation of all normal RR intervals for all 5-min segments).

pNN (percent difference between adjacent normal RR intervals greater than 20 ms, 30 ms, 50 ms, greater than 50 ms).

rMSSD (square root of the mean of the sum of the squared differences between adjacent normal RR intervals), SDANN (standard deviation of the mean of all normal RR intervals for 5-min segments), SDNNIDX (mean of the standard deviation of all normal RR intervals for all 5-min segments).

**Table 2:** HRV indices in the geometrical domain (all 5-min segments).

			**Smoothed centiles for Tri ind (All)**							**Smoothed centiles for Tri ind (Girls)**							**Smoothed centiles for Tri ind (Boys)**			
Months	n						Months	n						Months	n					
		3rd	10th	50th	90th	97th			3rd	10th	50th	90th	97th			3rd	10th	50th	90th	97th
0	260	11.7	14.1	20.5	28.5	32.9	0	117	10.9	13.4	19.8	28.3	33.1	0.0	143	12.0	14.8	21.2	28.2	31.4
6	205	12.4	14.9	21.1	28.5	32.1	6	95	12.6	14.7	20.3	27.7	31.8	6.0	110	12.4	15.2	21.7	28.8	32.0
12	198	12.8	15.5	22.2	29.6	33.1	12	86	13.3	15.6	21.3	28.5	32.3	12.0	112	12.8	15.7	22.7	30.4	33.9
18	187	12.9	16.1	23.6	32.1	35.9	18	82	13.1	16.0	22.9	30.8	34.6	18.0	105	12.9	16.3	24.2	32.9	36.9
24	182	12.7	16.5	25.5	35.7	40.5	24	80	12.0	16.0	25.0	34.4	38.6	24.0	102	13.0	16.9	26.2	36.4	41.1
Total	1032						Total	460						Total	572					
			**Smoothed centiles for TINN (ms) (All)**							**Smoothed centiles for TINN (ms) (Girls)**							**Smoothed centiles for TINN (ms) (Boys)**			
Months	n						Months	n						Months	n					
		3rd	10th	50th	90th	97th			3rd	10th	50th	90th	97th			3rd	10th	50th	90th	97th
0	260	183	221	320	446	513	0	117	170	209	310	443	516	0	143	189	231	331	442	493
6	205	193	233	330	445	502	6	95	196	230	318	433	497	6	110	195	238	339	451	501
12	198	199	242	346	463	518	12	86	208	243	333	446	505	12	112	199	245	355	475	529
18	187	201	251	369	501	562	18	82	206	250	357	481	541	18	105	202	254	378	515	577
24	182	199	258	399	558	633	24	80	189	250	390	538	604	24	102	204	265	409	569	643
Total	1032						Total	460						Total	572					
			**Smoothed centiles for X (ms) (All)**							**Smoothed centiles for X (ms) (Girls)**							**Smoothed centiles for X (ms) (Boys)**			
Months	n						Months	n						Months	n					
		3rd	10th	50th	90th	97th			3rd	10th	50th	90th	97th			3rd	10th	50th	90th	97th
0	260	396	418	472	539	575	0	117	392	415	471	537	571	0	143	398	420	474	540	574
6	205	375	405	476	551	585	6	95	373	403	472	545	578	6	110	376	408	480	555	588
12	198	371	407	490	575	610	12	86	370	405	483	564	599	12	112	371	409	496	582	616
18	187	382	422	515	610	650	18	82	384	421	506	596	635	18	105	381	425	523	620	659
24	182	409	451	551	657	704	24	80	414	451	539	639	686	24	102	406	453	560	669	715
Total	1032						Total	460						Total	572					
			**Smoothed centiles for Y (n) (All)**							**Smoothed centiles for Y (n) (Girls)**							**Smoothed centiles for Y (n) (Boys)**			
Months	n						Months	n						Months	n					
		3rd	10th	50th	90th	97th			3rd	10th	50th	90th	97th			3rd	10th	50th	90th	97th
0	260	2517	3651	6457	9798	11433	0	117	2494	3764	6798	10188	11756	0	143	2560	3587	6189	9422	11071
6	205	2513	3483	5913	8911	10472	6	95	2517	3613	6279	9369	10859	6	110	2501	3415	5672	8398	9807
12	198	2414	3235	5360	8195	9830	12	86	2439	3370	5705	8587	10082	12	112	2367	3161	5151	7706	9178
18	187	2219	2903	4792	7643	9498	18	82	2260	3034	5071	7835	9419	18	105	2161	2825	4630	7349	9182
24	182	1929	2491	4215	7260	9481	24	80	1982	2607	4382	7121	8875	24	102	1881	2408	4104	7322	9814
Total	1032						Total	460						Total	572					
			**Smoothed centiles for M (ms) (All)**							**Smoothed centiles for M (ms) (Girls)**							**Smoothed centiles for M (ms) (Boys)**			
Months	n						Months	n						Months	n					
		3rd	10th	50th	90th	97th			3rd	10th	50th	90th	97th			3rd	10th	50th	90th	97th
0	260	521	549	624	722	776	0	117	518	543	611	711	774	0	143	524	557	636	728	772
6	205	534	566	646	750	805	6	95	532	560	634	738	797	6	110	539	573	656	758	808
12	198	549	586	678	789	845	12	86	549	582	667	777	836	12	112	553	592	687	797	850
18	187	565	611	720	840	896	18	82	569	609	710	830	890	18	105	564	614	728	846	897
24	182	583	640	771	902	957	24	80	593	642	761	896	960	24	102	574	639	779	904	950
Total	1032						Total	460						Total	572					
			**Smoothed centiles for N (ms) (All)**							**Smoothed centiles for N (ms) (Girls)**							**Smoothed centiles for N (ms) (Boys)**			
Months	n						Months	n						Months	n					
		3rd	10th	50th	90th	97th			3rd	10th	50th	90th	97th			3rd	10th	50th	90th	97th
0	260	226	251	305	354	371	0	117	222	249	304	350	365	0	143	228	253	307	355	373
6	205	243	266	318	371	393	6	95	245	267	317	368	389	6	110	242	266	320	373	395
12	198	256	279	332	392	419	12	86	262	282	332	390	418	12	112	252	276	333	392	419
18	187	264	289	347	416	449	18	82	273	295	349	417	452	18	105	259	284	346	414	446
24	182	268	296	363	444	483	24	80	279	304	369	449	491	24	102	262	290	359	438	475
Total	1032						Total	460						Total	572					

HRV triangular index (integral of the density distribution divided by the maximum of the density distribution (Y)).

TINN (Triangular interpolation of the RR interval histogram width of a triangle using the minimum square difference).

**Table 3:** HRV indices in the frequency domain (all 5-min segments).

			**Smoothed centiles for Ptot (ms²/ Hz) (All)**							**Smoothed centiles for Ptot (ms²/ Hz) (Girls)**							**Smoothed centiles for Ptot (ms²/ Hz) (Boys)**			
Months	n						Months	n						Months	n					
		3rd	10th	50th	90th	97th			3rd	10th	50th	90th	97th			3rd	10th	50th	90th	97th
0	260	1266	1862	3712	7107	9723	0	117	1134	1662	3341	6449	8645	0	143	1399	2080	4081	7468	9957
6	205	1171	1714	3507	7243	10514	6	95	1171	1621	3172	6817	10598	6	110	1226	1835	3781	7585	10727
12	198	1106	1662	3607	8102	12435	12	86	1194	1628	3246	7669	12944	12	112	1105	1730	3865	8551	12824
18	187	1069	1705	4011	9693	15505	18	82	1201	1684	3564	9015	15709	18	105	1037	1763	4331	10353	16226
24	182	1061	1844	4716	11999	19692	24	80	1193	1788	4122	10838	18861	24	102	1022	1934	5178	12995	20939
Total	1032						Total	460						Total	572					
			**Smoothed centiles for ULF (ms²/ Hz) (All)**							**Smoothed centiles for ULF (ms²/ Hz) (Girls)**							**Smoothed centiles for ULF (ms²/ Hz) (Boys)**			
Months	n						Months	n						Months	n					
		3rd	10th	50th	90th	97th			3rd	10th	50th	90th	97th			3rd	10th	50th	90th	97th
0	260	473	806	1865	3895	5517	0	117	399	716	1729	3626	5017	0	143	549	895	1990	4080	5758
6	205	467	807	1956	4451	6741	6	95	511	793	1791	4250	6907	6	110	462	838	2077	4640	6869
12	198	451	801	2031	4908	7733	12	86	551	814	1815	4636	8047	12	112	407	813	2187	5174	7896
18	187	424	787	2091	5271	8502	18	82	520	780	1801	4788	8448	18	105	386	822	2320	5679	8832
24	182	387	765	2137	5537	9041	24	80	418	691	1750	4705	8106	24	102	398	863	2476	6159	9683
Total	1032						Total	460						Total	572					
			**Smoothed centiles for VLF (ms²/ Hz) (All)**							**Smoothed centiles for VLF (ms²/ Hz) (Girls)**							**Smoothed centiles for VLF (ms²/ Hz) (Boys)**			
Months	n						Months	n						Months	n					
		3rd	10th	50th	90th	97th			3rd	10th	50th	90th	97th			3rd	10th	50th	90th	97th
0	260	296	450	946	1915	2709	0	117	288	411	831	1785	2703	0	143	313	500	1052	1979	2640
6	205	241	350	690	1322	1815	6	95	242	326	616	1250	1816	6	110	253	380	753	1367	1792
12	198	218	314	605	1113	1487	12	86	217	300	561	1044	1408	12	112	228	331	638	1166	1548
18	187	227	342	688	1286	1722	18	82	212	333	667	1164	1473	18	105	238	352	708	1374	1905
24	182	267	433	938	1840	2517	24	80	229	424	931	1610	2010	24	102	282	443	960	1988	2860
Total	1032						Total	460						Total	572					
			**Smoothed centiles for LF (ms²/ Hz) (All)**							**Smoothed centiles for LF (ms²/ Hz) (Girls)**							**Smoothed centiles for LF (ms²/ Hz) (Boys)**			
Months	n						Months	n						Months	n					
		3rd	10th	50th	90th	97th			3rd	10th	50th	90th	97th			3rd	10th	50th	90th	97th

0	260	137	203	435	980	1513	0	117	121	174	369	863	1385	0	143	167	241	494	1055	1581
6	205	137	198	416	953	1505	6	95	125	177	370	877	1434	6	110	161	225	453	1002	1556
12	198	135	201	445	1058	1703	12	86	128	188	411	988	1618	12	112	150	219	472	1109	1778
18	187	130	214	523	1298	2110	18	82	130	208	491	1195	1940	18	105	133	222	548	1375	2244
24	182	122	236	648	1669	2723	24	80	131	237	610	1498	2395	24	102	111	235	684	1799	2954
Total	1032						Total	460						Total	572					
			**Smoothed centiles for HF (ms²/ Hz) (All)**							**Smoothed centiles for HF (ms²/ Hz) (Girls)**							**Smoothed centiles for HF (ms²/ Hz) (Boys)**			
Months	n						Months	n						Months	n					
		3rd	10th	50th	90th	97th			3rd	10th	50th	90th	97th			3rd	10th	50th	90th	97th

0	260	66.7	100.0	247.5	779.0	1554.4	0	117	63.5	88.6	202.5	625.8	1247.8	0	143	72.8	115.8	294.4	876.2	1676.1
6	205	67.5	102.0	263.0	902.1	1916.2	6	95	62.6	91.4	228.6	805.1	1783.7	6	110	74.3	114.4	295.5	968.7	1961.0
12	198	68.8	114.8	343.5	1369.3	3192.8	12	86	64.6	106.7	315.6	1259.2	2963.8	12	112	74.1	123.2	367.8	1461.7	3385.3
18	187	70.6	138.5	489.7	2185.6	5398.1	18	82	69.7	134.9	464.4	1993.1	4801.1	18	105	72.3	142.1	510.2	2349.2	5931.7
24	182	73.0	172.9	700.1	3342.2	8508.2	24	80	77.7	175.6	673.3	2998.8	7275.5	24	102	68.8	171.0	723.0	3632.9	9605.0
Total	1032						Total	460						Total	572					
			**Smoothed centiles for LF/HF (All)**							**Smoothed centiles for LF/HF (Girls)**							**Smoothed centiles for LF/HF (Boys)**			
Months	n						Months	n						Months	n					
		3rd	10th	50th	90th	97th			3rd	10th	50th	90th	97th			3rd	10th	50th	90th	97th
0	260	0.99	1.46	2.69	4.34	5.26	0	117	0.94	1.45	2.79	4.60	5.61	0	143	1.04	1.47	2.61	4.11	4.93
6	205	0.89	1.24	2.23	3.79	4.85	6	95	0.91	1.29	2.36	3.91	4.88	6	110	0.88	1.20	2.13	3.67	4.76
12	198	0.77	1.04	1.84	3.29	4.39	12	86	0.82	1.11	1.96	3.33	4.27	12	112	0.74	0.98	1.75	3.23	4.42
18	187	0.64	0.86	1.53	2.84	3.88	18	82	0.68	0.91	1.62	2.86	3.79	18	105	0.62	0.82	1.47	2.80	3.92
24	182	0.50	0.70	1.30	2.43	3.33	24	80	0.48	0.68	1.31	2.49	3.43	24	102	0.52	0.71	1.29	2.38	3.25
Total	1032						Total	460						Total	572					

Ptot (total power), ULF (ultra-low frequencies), VLF (very low frequencies), LF (low frequencies), and HF (high frequencies).

**Table 4:** HRV indices by Poincaré plot (all 5-min segments).

			**Smoothed centiles for Centroïd (ms) (All)**							**Smoothed centiles for Centroïd (ms) (Girls)**							**Smoothed centiles for Centroïd (ms) (Boys)**			
Months	n						Months	n						Months	n					
		3rd	10th	50th	90th	97th			3rd	10th	50th	90th	97th			3rd	10th	50th	90th	97th
0	260	420	433	467	515	544	0	117	420	430	460	508	539	0	143	421	435	472	521	548
6	205	425	442	483	532	556	6	95	423	439	477	524	549	6	110	428	445	488	536	558
12	198	435	455	504	558	583	12	86	432	452	499	551	575	12	112	436	459	509	561	583
18	187	448	473	532	595	623	18	82	448	472	527	589	618	18	105	447	475	536	597	622
24	182	464	494	565	642	677	24	80	470	496	560	638	677	24	102	461	494	569	644	674
Total	1032						Total	460						Total	572					
			**Smoothed centiles for SD1 (ms) (All)**							**Smoothed centiles for SD1 (ms) (Girls)**							**Smoothed centiles for SD1 (ms) (Boys)**			
Months	n						Months	n						Months	n					
		3rd	10th	50th	90th	97th			3rd	10th	50th	90th	97th			3rd	10th	50th	90th	97th
0	260	7.41	9.14	14.64	25.27	33.89	0	117	7.21	8.58	13.24	23.64	33.51	0	143	7.95	9.88	15.79	26.43	34.53
6	205	8.05	9.86	15.77	27.65	37.71	6	95	7.62	9.24	14.59	25.76	35.65	6	110	8.70	10.59	16.71	29.02	39.50
12	198	8.52	10.76	18.11	33.22	46.25	12	86	8.05	10.14	17.01	31.19	43.53	12	112	9.12	11.42	19.00	34.72	48.39
18	187	8.83	11.83	21.71	42.03	59.59	18	82	8.50	11.28	20.53	40.02	57.24	18	105	9.22	12.37	22.66	43.46	61.12
24	182	8.98	13.07	26.51	54.00	77.60	24	80	8.97	12.65	25.11	52.13	76.62	24	102	8.98	13.44	27.68	55.28	77.73
Total	1032						Total	460						Total	572					
			**Smoothed centiles for SD2 (ms) (All)**							**Smoothed centiles for SD2 (ms) (Girls)**							**Smoothed centiles for SD2 (ms) (Boys)**			
Months	n						Months	n						Months	n					
		3rd	10th	50th	90th	97th			3rd	10th	50th	90th	97th			3rd	10th	50th	90th	97th
0	260	52.08	62.74	89.66	123.07	140.36	0	117	49.50	59.68	86.19	121.23	140.64	0	143	52.27	65.32	94.21	122.09	133.11
6	205	59.46	70.42	97.69	130.71	147.41	6	95	59.66	69.11	93.80	126.68	145.05	6	110	60.31	72.27	100.89	133.07	148.12
12	198	63.53	75.83	106.03	141.78	159.44	12	86	65.24	75.32	101.64	136.58	156.04	12	112	64.29	77.29	108.98	145.71	163.35
18	187	64.36	79.02	114.78	156.40	176.56	18	82	66.29	78.39	109.81	151.05	173.72	18	105	64.22	80.38	118.43	159.92	178.72
24	182	61.92	79.98	123.84	174.40	198.60	24	80	62.81	78.27	118.20	169.92	197.90	24	102	60.13	81.57	129.28	175.78	194.32
Total	1032						Total	460						Total	572					
			**Smoothed centiles for SD1/SD2 ratio (All)**							**Smoothed centiles for SD1/SD2 ratio (Girls)**							**Smoothed centiles for SD1/SD2 ratio (Boys)**			
Months	n						Months	n						Months	n					
		3rd	10th	50th	90th	97th			3rd	10th	50th	90th	97th			3rd	10th	50th	90th	97th
0	260	0.095	0.113	0.168	0.260	0.325	0	117	0.089	0.107	0.161	0.253	0.320	0	143	0.102	0.120	0.173	0.264	0.330
6	205	0.096	0.113	0.165	0.259	0.330	6	95	0.094	0.110	0.158	0.247	0.316	6	110	0.098	0.116	0.171	0.267	0.338
12	198	0.098	0.117	0.173	0.275	0.353	12	86	0.099	0.115	0.167	0.263	0.338	12	112	0.098	0.118	0.179	0.284	0.361
18	187	0.102	0.125	0.192	0.310	0.395	18	82	0.101	0.123	0.188	0.301	0.385	18	105	0.102	0.126	0.197	0.315	0.400
24	182	0.107	0.136	0.222	0.361	0.456	24	80	0.103	0.133	0.220	0.362	0.459	24	102	0.110	0.139	0.223	0.360	0.453
Total	1032						Total	460						Total	572					
			**Smoothed centiles for SD1nu (%) (All)**							**Smoothed centiles for SD1nu (%) (Girls)**							**Smoothed centiles for SD1nu (%) (Boys)**			
Months	n						Months	n						Months	n					
		3rd	10th	50th	90th	97th			3rd	10th	50th	90th	97th			3rd	10th	50th	90th	97th
0	260	1.68	2.03	3.12	5.14	6.69	0	117	1.62	1.92	2.88	4.84	6.54	0	143	1.80	2.18	3.32	5.35	6.89
6	205	1.76	2.13	3.27	5.48	7.25	6	95	1.69	2.01	3.07	5.16	6.91	6	110	1.88	2.26	3.44	5.71	7.57
12	198	1.82	2.24	3.58	6.22	8.41	12	86	1.74	2.14	3.40	5.89	7.97	12	112	1.93	2.35	3.73	6.48	8.79
18	187	1.86	2.38	4.05	7.39	10.17	18	82	1.79	2.28	3.87	7.06	9.74	18	105	1.94	2.47	4.19	7.64	10.53
24	182	1.87	2.53	4.68	8.95	12.53	24	80	1.83	2.45	4.48	8.64	12.20	24	102	1.90	2.60	4.83	9.20	12.79
Total	1032						Total	460						Total	572					
			**Smoothed centiles for SD2nu (%) (All)**							**Smoothed centiles for SD2nu (%) (Girls)**							**Smoothed centiles for SD2nu (%) (Boys)**			
Months	n						Months	n						Months	n					
		3rd	10th	50th	90th	97th			3rd	10th	50th	90th	97th			3rd	10th	50th	90th	97th
0	260	11.96	13.98	19.04	25.28	28.48	0	117	11.42	13.43	18.57	25.11	28.60	0	143	12.06	14.43	19.72	24.94	27.06
6	205	13.11	15.16	20.16	25.96	28.77	6	95	13.21	15.02	19.62	25.37	28.35	6	110	13.12	15.40	20.65	26.20	28.62
12	198	13.46	15.71	21.03	26.86	29.49	12	86	13.80	15.73	20.46	26.00	28.69	12	112	13.45	15.85	21.45	27.44	30.09
18	187	13.00	15.63	21.68	27.97	30.66	18	82	13.22	15.57	21.07	27.00	29.65	18	105	13.05	15.79	22.10	28.66	31.47
24	182	11.74	14.91	22.08	29.28	32.27	24	80	11.45	14.54	21.47	28.37	31.20	24	102	11.92	15.23	22.62	29.86	32.76
Total	1032						Total	460						Total	572					

SD1 and SD2 (standard deviation of the distances of the RR intervals from the y = x line and the y = −x + 2 mean (RR) line, respectively).

**Table 5:** HRV indices by an empirical mode decomposition (all 5-min segments).

			**Smoothed centiles for pLF1 (All)**							**Smoothed centiles for pLF1 (Girls)**							**Smoothed centiles for pLF1 (Boys)**			
Months	n						Months	n						Months	n					
		3rd	10th	50th	90th	97th			3rd	10th	50th	90th	97th			3rd	10th	50th	90th	97th
0	260	76.2	112.5	237.6	518.3	781.7	0	117	68.8	97.4	200.7	457.4	726.8	0	143	91.0	133.0	270.8	555.0	803.5
6	205	83.4	119.1	245.2	541.9	833.6	6	95	81.2	110.8	219.0	495.9	795.6	6	110	93.3	132.1	266.3	572.0	866.1
12	198	86.3	128.5	277.8	631.1	980.6	12	86	88.6	124.8	255.3	582.0	930.6	12	112	90.5	135.9	295.3	666.7	1029.3
18	187	85.0	140.7	335.7	787.0	1224.5	18	82	91.2	139.6	310.0	716.9	1133.5	18	105	82.6	144.3	357.4	837.8	1291.3
24	182	79.5	155.8	418.4	1007.9	1562.5	24	80	89.0	155.1	382.4	898.9	1401.9	24	102	69.6	157.3	452.7	1085.7	1652.6
Total	1032						Total	460						Total	572					
			**Smoothed centiles for pLF2 (All)**							**Smoothed centiles for pLF2 (Girls)**							**Smoothed centiles for pLF2 (Boys)**			
Months	n						Months	n						Months	n					
		3rd	10th	50th	90th	97th			3rd	10th	50th	90th	97th			3rd	10th	50th	90th	97th
0	260	85.8	128.3	263.4	525.1	738.4	0	117	78.5	114.4	234.1	493.6	736.6	0	143	93.0	144.6	293.5	532.4	694.4
6	205	89.4	129.1	255.5	499.0	695.8	6	95	88.8	121.6	231.6	465.7	673.9	6	110	94.4	139.5	276.3	518.6	699.9
12	198	92.4	137.7	279.8	545.7	754.5	12	86	95.5	134.4	259.0	502.0	700.5	12	112	94.6	143.6	296.4	579.4	799.5
18	187	94.6	154.0	336.6	665.8	915.2	18	82	98.8	153.2	316.6	602.8	816.7	18	105	93.6	156.9	353.1	713.9	992.0
24	182	96.0	178.0	425.3	857.8	1176.1	24	80	98.5	177.7	403.7	767.0	1021.2	24	102	91.4	179.2	446.6	922.2	1277.6
Total	1032						Total	460						Total	572					
			**Smoothed centiles for pHF1 (All)**							**Smoothed centiles for pHF1 (Girls)**							**Smoothed centiles for pHF1 (Boys)**			
Months	n						Months	n						Months	n					
		3rd	10th	50th	90th	97th			3rd	10th	50th	90th	97th			3rd	10th	50th	90th	97th
0	260	60.5	88.7	197.5	497.7	837.9	0	117	60.3	79.8	161.0	427.1	785.2	0	143	68.7	104.0	230.6	546.2	890.1
6	205	54.7	78.0	172.3	460.5	824.3	6	95	51.7	70.1	145.6	391.4	731.6	6	110	64.1	89.9	193.8	516.9	940.6
12	198	52.4	79.4	197.1	613.3	1214.7	12	86	49.8	73.5	175.8	539.8	1085.6	12	112	59.5	87.3	213.0	677.1	1363.4
18	187	53.4	92.7	272.1	957.5	2013.2	18	82	54.5	90.1	251.9	874.1	1851.0	18	105	54.8	96.3	287.8	1024.4	2153.1
24	182	57.8	117.9	396.4	1489.6	3211.2	24	80	65.8	119.7	373.2	1390.5	3019.5	24	102	50.0	116.6	418.0	1559.1	3311.0
Total	1032						Total	460						Total	572					
			**Smoothed centiles for pHF2 (All)**							**Smoothed centiles for pHF2 (Girls)**							**Smoothed centiles for pHF2 (Boys)**			
Months	n						Months	n						Months	n					
		3rd	10th	50th	90th	97th			3rd	10th	50th	90th	97th			3rd	10th	50th	90th	97th
0	260	57.0	88.9	213.5	537.5	813.3	0	117	57.2	82.0	178.4	405.4	509.2	0	143	61.7	100.5	247.5	638.9	1065.1
6	205	54.5	86.5	238.6	890.3	2045.0	6	95	48.5	76.4	210.0	803.8	1907.7	6	110	65.4	99.4	261.2	963.5	2221.3
12	198	56.5	100.6	333.5	1510.0	3843.1	12	86	49.8	90.6	302.2	1365.8	3506.6	12	112	67.4	112.3	357.3	1643.6	4235.1
18	187	63.1	131.5	499.2	2403.4	6227.1	18	82	61.2	124.8	456.0	2097.5	5323.5	18	105	67.8	139.0	534.5	2672.7	7088.4
24	182	74.3	178.9	734.1	3560.7	9171.2	24	80	82.4	178.8	669.7	2990.9	7338.4	24	102	66.4	179.6	793.2	4053.5	10790.1
Total	1032						Total	460						Total	572					
			**Smoothed centiles for IMAI1 (All)**							**Smoothed centiles for IMAI1 (Girls)**							**Smoothed centiles for IMAI1 (Boys)**			
Months	n						Months	n						Months	n					
		3rd	10th	50th	90th	97th			3rd	10th	50th	90th	97th			3rd	10th	50th	90th	97th
0	260	0.383	0.501	0.819	1.262	1.522	0	117	0.389	0.517	0.857	1.328	1.604	0	143	0.384	0.493	0.787	1.202	1.449
6	205	0.382	0.487	0.774	1.192	1.451	6	95	0.402	0.513	0.807	1.220	1.468	6	110	0.370	0.469	0.747	1.163	1.426
12	198	0.349	0.442	0.702	1.100	1.359	12	86	0.372	0.469	0.729	1.106	1.342	12	112	0.333	0.423	0.681	1.089	1.362
18	187	0.284	0.367	0.604	0.986	1.246	18	82	0.298	0.383	0.621	0.985	1.224	18	105	0.275	0.355	0.591	0.983	1.258
24	182	0.189	0.262	0.479	0.849	1.113	24	80	0.179	0.257	0.485	0.858	1.115	24	102	0.196	0.265	0.475	0.843	1.114
Total	1032						Total	460						Total	572					
			**Smoothed centiles for IMAI2 (All)**							**Smoothed centiles for IMAI2 (Girls)**							**Smoothed centiles for IMAI2 (Boys)**			
Months	n						Months	n						Months	n					
		3rd	10th	50th	90th	97th			3rd	10th	50th	90th	97th			3rd	10th	50th	90th	97th
0	260	0.356	0.513	0.962	1.682	2.167	0	117	0.372	0.548	1.054	1.859	2.402	0	143	0.346	0.492	0.899	1.513	1.902
6	205	0.352	0.482	0.851	1.435	1.824	6	95	0.377	0.516	0.909	1.518	1.919	6	110	0.337	0.460	0.809	1.354	1.712
12	198	0.320	0.430	0.746	1.256	1.605	12	86	0.344	0.458	0.780	1.281	1.613	12	112	0.306	0.410	0.718	1.228	1.586
18	187	0.260	0.357	0.645	1.142	1.505	18	82	0.274	0.375	0.667	1.145	1.479	18	105	0.252	0.345	0.628	1.137	1.524
24	182	0.172	0.263	0.551	1.095	1.526	24	80	0.165	0.265	0.570	1.112	1.520	24	102	0.176	0.262	0.538	1.080	1.526
Total	1032						Total	460						Total	572					

Nonlinear and non-stationary time series are decomposed into a limited number of oscillatory components (modes), pLF1, pLF2, pHF1, and pHF2 (Low and high frequencies power associated to the selected mode), IMAI1 and IMAI2 (ratios between low and high frequency indices).

**Table 6:** HRV indices by a fractal analysis (all 5-min segments).

			**Smoothed centiles for alpha1 DFA (All)**							**Smoothed centiles for alpha1 DFA (Girls)**							**Smoothed centiles for alpha1 DFA (Boys)**			
Months	n						Months	n						Months	n					
-		3rd	10th	50th	90th	97th			3rd	10th	50th	90th	97th			3rd	10th	50th	90th	97th
0	260	0.96	1.04	1.20	1.35	1.40	0	117	0.95	1.04	1.22	1.36	1.41	0	143	0.96	1.04	1.19	1.33	1.38
6	205	0.92	0.99	1.15	1.31	1.37	6	95	0.92	1.00	1.17	1.32	1.37	6	110	0.92	0.99	1.14	1.30	1.36
12	198	0.87	0.94	1.10	1.27	1.34	12	86	0.88	0.95	1.11	1.28	1.34	12	112	0.87	0.93	1.09	1.26	1.34
18	187	0.81	0.88	1.05	1.23	1.32	18	82	0.81	0.89	1.06	1.24	1.32	18	105	0.82	0.88	1.04	1.23	1.31
24	182	0.75	0.82	1.00	1.20	1.29	24	80	0.73	0.81	1.01	1.21	1.31	24	102	0.76	0.83	1.00	1.19	1.28
Total	1032						Total	460						Total	572					
			**Smoothed centiles for alpha2 DFA (All)**							**Smoothed centiles for alpha2 DFA (Girls)**							**Smoothed centiles for alpha2 DFA (Boys)**			
Months	n						Months	n						Months	n					
-		3rd	10th	50th	90th	97th			3rd	10th	50th	90th	97th			3rd	10th	50th	90th	97th
0	260	0.87	0.93	1.05	1.16	1.20	0	117	0.88	0.94	1.07	1.18	1.22	0	143	0.87	0.92	1.03	1.14	1.18
6	205	0.80	0.84	0.94	1.04	1.09	6	95	0.80	0.85	0.95	1.05	1.10	6	110	0.79	0.84	0.93	1.03	1.07
12	198	0.75	0.79	0.88	0.98	1.03	12	86	0.75	0.79	0.89	0.98	1.03	12	112	0.75	0.79	0.88	0.98	1.02
18	187	0.74	0.77	0.87	0.98	1.03	18	82	0.73	0.78	0.88	0.98	1.02	18	105	0.73	0.77	0.87	0.98	1.03
24	182	0.75	0.80	0.91	1.03	1.09	24	80	0.75	0.80	0.92	1.02	1.06	24	102	0.75	0.79	0.90	1.03	1.10
Total	1032						Total	460						Total	572					
			**Smoothed centiles for H DFA (All)**							**Smoothed centiles for H DFA (Girls)**							**Smoothed centiles for H DFA (Boys)**			
Months	n						Months	n						Months	n					
-		3rd	10th	50th	90th	97th			3rd	10th	50th	90th	97th			3rd	10th	50th	90th	97th
0	260	0.94	0.97	1.04	1.11	1.14	0	117	0.95	0.98	1.04	1.12	1.15	0	143	0.94	0.97	1.03	1.10	1.14
6	205	0.93	0.95	1.01	1.07	1.10	6	95	0.92	0.95	1.01	1.07	1.09	6	110	0.93	0.95	1.00	1.07	1.10
12	198	0.91	0.93	0.98	1.04	1.07	12	86	0.90	0.93	0.98	1.04	1.06	12	112	0.91	0.93	0.98	1.05	1.08
18	187	0.89	0.91	0.97	1.03	1.06	18	82	0.89	0.91	0.97	1.02	1.05	18	105	0.90	0.92	0.97	1.03	1.07
24	182	0.88	0.90	0.96	1.03	1.07	24	80	0.89	0.91	0.96	1.03	1.06	24	102	0.88	0.90	0.96	1.03	1.07
Total	1032						Total	460						Total	572					
			**Smoothed centiles for H Higuchi (All)**							**Smoothed centiles for H Higuchi (Girls)**							**Smoothed centiles for H Higuchi (Boys)**			
Months	n						Months	n						Months	n					
-		3rd	10th	50th	90th	97th			3rd	10th	50th	90th	97th			3rd	10th	50th	90th	97th
0	260	1.45	1.49	1.61	1.75	1.82	0	117	1.45	1.50	1.61	1.75	1.81	0	143	1.45	1.49	1.60	1.75	1.82
6	205	1.52	1.57	1.70	1.83	1.89	6	95	1.52	1.57	1.69	1.83	1.89	6	110	1.51	1.57	1.70	1.84	1.89
12	198	1.57	1.63	1.76	1.90	1.95	12	86	1.57	1.62	1.75	1.89	1.95	12	112	1.57	1.63	1.77	1.90	1.95
18	187	1.61	1.67	1.80	1.94	2.00	18	82	1.60	1.66	1.79	1.93	1.99	18	105	1.62	1.68	1.81	1.94	2.00
24	182	1.64	1.69	1.81	1.96	2.03	24	80	1.63	1.68	1.80	1.95	2.03	24	102	1.65	1.70	1.82	1.96	2.02
Total	1032						Total	460						Total	572					
			**Smoothed centiles for H Katz (All)**							**Smoothed centiles for H Katz (Girls)**							**Smoothed centiles for H Katz (Boys)**			
Months	n						Months	n						Months	n					
-		3rd	10th	50th	90th	97th			3rd	10th	50th	90th	97th			3rd	10th	50th	90th	97th
0	260	1.26	1.30	1.41	1.56	1.65	0	117	1.26	1.29	1.39	1.54	1.63	0	143	1.27	1.31	1.42	1.57	1.65
6	205	1.30	1.34	1.44	1.61	1.72	6	95	1.29	1.32	1.43	1.58	1.68	6	110	1.31	1.35	1.45	1.62	1.74
12	198	1.32	1.36	1.48	1.68	1.81	12	86	1.31	1.35	1.47	1.65	1.75	12	112	1.33	1.37	1.50	1.70	1.83
18	187	1.33	1.38	1.54	1.77	1.91	18	82	1.32	1.37	1.52	1.73	1.86	18	105	1.34	1.39	1.55	1.79	1.95
24	182	1.32	1.40	1.59	1.87	2.04	24	80	1.32	1.39	1.57	1.83	1.98	24	102	1.32	1.40	1.61	1.90	2.07
Total	1032						Total	460						Total	572					
			**Smoothed centiles for Hurst (All)**							**Smoothed centiles for Hurst (Girls)**							**Smoothed centiles for Hurst (Boys)**			
Months	n						Months	n						Months	n					
-		3rd	10th	50th	90th	97th			3rd	10th	50th	90th	97th			3rd	10th	50th	90th	97th
0	260	0.198	0.253	0.370	0.476	0.515	0	117	0.202	0.256	0.372	0.478	0.518	0	143	0.196	0.251	0.368	0.474	0.514
6	205	0.142	0.191	0.304	0.421	0.471	6	95	0.146	0.198	0.313	0.424	0.468	6	110	0.139	0.186	0.297	0.416	0.470
12	198	0.097	0.144	0.254	0.376	0.433	12	86	0.102	0.152	0.267	0.381	0.429	12	112	0.093	0.137	0.244	0.370	0.430
18	187	0.063	0.109	0.220	0.343	0.400	18	82	0.070	0.119	0.233	0.351	0.401	18	105	0.057	0.102	0.210	0.335	0.394
24	182	0.039	0.088	0.202	0.321	0.372	24	80	0.050	0.099	0.213	0.332	0.384	24	102	0.032	0.081	0.194	0.311	0.362
Total	1032						Total	460						Total	572					
			**Smoothed centiles for Beta 1/f Slope (All)**							**Smoothed centiles for Beta 1/f Slope (Girls)**							**Smoothed centiles for Beta 1/f Slope (Boys)**			
Months	n						Months	n						Months	n					
-		3rd	10th	50th	90th	97th			3rd	10th	50th	90th	97th			3rd	10th	50th	90th	97th
0	260	-1.63	-1.49	-1.15	-0.73	-0.51	0	117	-1.65	-1.49	-1.14	-0.78	-0.62	0	143	-1.62	-1.49	-1.15	-0.71	-0.48
6	205	-1.80	-1.62	-1.21	-0.76	-0.55	6	95	-1.78	-1.61	-1.20	-0.76	-0.56	6	110	-1.80	-1.62	-1.21	-0.78	-0.58
12	198	-1.91	-1.71	-1.23	-0.74	-0.52	12	86	-1.89	-1.69	-1.23	-0.71	-0.47	12	112	-1.93	-1.71	-1.24	-0.78	-0.59
18	187	-1.98	-1.75	-1.22	-0.67	-0.43	18	82	-1.96	-1.74	-1.22	-0.64	-0.36	18	105	-1.99	-1.75	-1.22	-0.71	-0.50
24	182	-2.00	-1.75	-1.17	-0.55	-0.27	24	80	-2.00	-1.75	-1.17	-0.53	-0.23	24	102	-2.01	-1.75	-1.16	-0.57	-0.32
Total	1032						Total	460						Total	572					

DFA (detrended fluctuation analysis to quantify the degree of self-similarity (fractuality) of the RR signal.

α1 and α2 (the slope of short-and long-term fluctuations, respectively).

Higuchi and Katz algorithms and H (Hurst exponent) measure the self-similarity of the RR signal.

ß 1/f slope index (calculated on the PSD plotted on a log-log scale from 10^-4^ to 10^-2^ Hz).

**Table 7:** HRV indices by a Chaos and DC or AC analysis (all 5-min segments).

			**Smoothed centiles for Skewness (All)**							**Smoothed centiles for Skewness (Girls)**							**Smoothed centiles for Skewness (Boys)**			
Months	n						Months	n						Months	n					
-		3rd	10th	50th	90th	97th			3rd	10th	50th	90th	97th			3rd	10th	50th	90th	97th
0	260	-0.292	-0.116	0.389	1.194	1.732	0	117	-0.280	-0.106	0.396	1.202	1.748	0	143	-0.300	-0.123	0.383	1.187	1.726
6	205	-0.179	-0.015	0.472	1.289	1.862	6	95	-0.181	-0.020	0.449	1.204	1.712	6	110	-0.174	-0.008	0.493	1.354	1.974
12	198	-0.151	0.025	0.530	1.339	1.890	12	86	-0.144	0.024	0.498	1.228	1.700	12	112	-0.151	0.030	0.557	1.422	2.025
18	187	-0.209	0.002	0.562	1.344	1.814	18	82	-0.167	0.026	0.545	1.276	1.712	18	105	-0.233	-0.010	0.577	1.389	1.881
24	182	-0.351	-0.082	0.569	1.303	1.636	24	80	-0.250	-0.014	0.588	1.348	1.747	24	102	-0.419	-0.127	0.552	1.258	1.543
Total	1032						Total	460						Total	572					
			**Smoothed centiles for Kurtosis (All)**							**Smoothed centiles for Kurtosis (Girls)**							**Smoothed centiles for Kurtosis (Boys)**			
Months	n						Months	n						Months	n					
-		3rd	10th	50th	90th	97th			3rd	10th	50th	90th	97th			3rd	10th	50th	90th	97th
0	260	2.84	3.41	5.82	13.97	25.19	0	117	2.66	3.27	5.94	15.63	30.27	0	143	3.07	3.56	5.66	13.00	23.37
6	205	3.31	3.97	6.45	13.20	20.95	6	95	3.19	3.91	6.43	12.81	20.09	6	110	3.44	4.05	6.46	13.56	22.26
12	198	3.50	4.19	6.62	12.23	17.59	12	86	3.42	4.17	6.58	11.07	14.28	12	112	3.58	4.24	6.68	13.06	19.97
18	187	3.42	4.09	6.34	11.07	15.07	18	82	3.34	4.08	6.39	10.39	12.77	18	105	3.49	4.11	6.33	11.52	16.53
24	182	3.06	3.65	6.23	9.72	13.42	24	80	2.97	3.63	5.86	10.78	15.58	24	102	3.16	3.69	5.42	8.93	11.92
Total	1032						Total	460						Total	572					
			**Smoothed centiles for Lyapunov exponent (All)**							**Smoothed centiles for Lyapunov exponent (Girls)**							**Smoothed centiles for Lyapunov exponent (Boys)**			
Months	n						Months	n						Months	n					
-		3rd	10th	50th	90th	97th			3rd	10th	50th	90th	97th			3rd	10th	50th	90th	97th
0	260	0.136	0.161	0.233	0.347	0.422	0	117	0.135	0.155	0.215	0.322	0.404	0	143	0.143	0.171	0.248	0.363	0.437
6	205	0.130	0.152	0.217	0.323	0.395	6	95	0.125	0.147	0.207	0.302	0.365	6	110	0.137	0.159	0.225	0.336	0.417
12	198	0.129	0.153	0.222	0.328	0.397	12	86	0.124	0.149	0.216	0.310	0.365	12	112	0.134	0.158	0.227	0.341	0.420
18	187	0.132	0.164	0.249	0.364	0.429	18	82	0.130	0.161	0.242	0.346	0.403	18	105	0.134	0.168	0.256	0.375	0.446
24	182	0.139	0.185	0.298	0.428	0.490	24	80	0.143	0.183	0.284	0.412	0.479	24	102	0.136	0.188	0.310	0.440	0.496
Total	1032						Total	460						Total	572					
			**Smoothed centiles for Acceleration Capacity (AC) (All)**							**Smoothed centiles for Acceleration Capacity (AC) (Girls)**							**Smoothed centiles for Acceleration Capacity (AC) (Boys)**			
Months	n						Months	n						Months	n					
-		3rd	10th	50th	90th	97th			3rd	10th	50th	90th	97th			3rd	10th	50th	90th	97th
0	260	-5.93	-5.10	-3.38	-1.94	-1.45	0	117	-5.89	-4.98	-3.18	-1.79	-1.36	0	143	-5.90	-5.16	-3.57	-2.10	-1.56
6	205	-7.18	-6.28	-4.35	-2.55	-1.86	6	95	-7.17	-6.22	-4.22	-2.44	-1.79	6	110	-7.13	-6.30	-4.47	-2.65	-1.91
12	198	-8.43	-7.42	-5.19	-3.03	-2.17	12	86	-8.43	-7.39	-5.11	-2.97	-2.16	12	112	-8.38	-7.43	-5.28	-3.08	-2.15
18	187	-9.69	-8.52	-5.92	-3.39	-2.39	18	82	-9.70	-8.50	-5.87	-3.41	-2.47	18	105	-9.65	-8.53	-5.99	-3.39	-2.29
24	182	-10.97	-9.58	-6.54	-3.64	-2.50	24	80	-10.96	-9.53	-6.48	-3.73	-2.72	24	102	-10.94	-9.61	-6.61	-3.58	-2.32
Total	1032						Total	460						Total	572					
			**Smoothed centiles for Deceleration Capacity (DC) (All)**							**Smoothed centiles for Deceleration Capacity (DC) (Girls)**							**Smoothed centiles for Deceleration Capacity (DC) (Boys)**			
Months	n						Months	n						Months	n					
-		3rd	10th	50th	90th	97th			3rd	10th	50th	90th	97th			3rd	10th	50th	90th	97th
0	260	1.70	2.30	3.73	5.31	6.04	0	117	1.49	2.09	3.50	5.08	5.82	0	143	1.91	2.50	3.91	5.49	6.22
6	205	2.13	2.83	4.52	6.41	7.29	6	95	2.33	2.89	4.33	6.17	7.18	6	110	2.05	2.86	4.68	6.47	7.20
12	198	2.45	3.28	5.26	7.41	8.39	12	86	2.77	3.44	5.11	7.19	8.33	12	112	2.25	3.24	5.39	7.43	8.22
18	187	2.64	3.65	5.96	8.34	9.36	18	82	2.82	3.74	5.85	8.15	9.25	18	105	2.53	3.63	6.05	8.36	9.27
24	182	2.70	3.92	6.61	9.17	10.18	24	80	2.48	3.77	6.54	9.05	9.96	24	102	2.88	4.04	6.66	9.28	10.36
Total	1032						Total	460						Total	572					

Skewness, Kurtosis and Lyapunov exponent used in nonlinear analysis of physiological signals for detecting chaos.

DC (deceleration capacity calculated by the difference between the mean of the 2 beats following deceleration and the mean of the 2 beats before deceleration).

AC (acceleration capacity calculated by detecting all sequences that decrease).

**Table 8:** Entropy analysis (all 5-min segments).

			**Smoothed centiles for AppEn (approximate entropy) (All)**							**Smoothed centiles for AppEn (approximate entropy) (Girls)**							**Smoothed centiles for AppEn (approximate entropy) (Boys)**			
Months	n						Months	n						Months	n					
		3rd	10th	50th	90th	97th			3rd	10th	50th	90th	97th			3rd	10th	50th	90th	97th
0	260	0.72	0.78	0.94	1.13	1.23	0	117	0.70	0.77	0.94	1.11	1.18	0	143	0.73	0.79	0.95	1.14	1.25
6	205	0.90	0.95	1.09	1.23	1.30	6	95	0.89	0.95	1.08	1.21	1.27	6	110	0.91	0.96	1.10	1.24	1.31
12	198	1.00	1.06	1.17	1.29	1.34	12	86	1.00	1.05	1.16	1.28	1.33	12	112	1.01	1.06	1.19	1.30	1.35
18	187	1.04	1.09	1.21	1.32	1.36	18	82	1.04	1.09	1.19	1.31	1.36	18	105	1.04	1.09	1.22	1.33	1.37
24	182	1.00	1.05	1.18	1.31	1.36	24	80	1.01	1.05	1.16	1.30	1.36	24	102	0.99	1.06	1.19	1.31	1.36
Total	1032						Total	460						Total	572					
			**Smoothed centiles for SampEn (Sample entropy) (All)**							**Smoothed centiles for SampEn (Sample entropy) (Girls)**							**Smoothed centiles for SampEn (Sample entropy) (Boys)**			
Months	n						Months	n						Months	n					
		3rd	10th	50th	90th	97th			3rd	10th	50th	90th	97th			3rd	10th	50th	90th	97th
0	260	0.50	0.56	0.72	0.93	1.06	0	117	0.51	0.56	0.71	0.89	0.99	0	143	0.50	0.56	0.73	0.96	1.09
6	205	0.74	0.81	0.97	1.17	1.28	6	95	0.75	0.81	0.96	1.15	1.25	6	110	0.74	0.81	0.98	1.19	1.30
12	198	0.89	0.95	1.11	1.31	1.42	12	86	0.89	0.95	1.10	1.30	1.41	12	112	0.88	0.95	1.12	1.33	1.43
18	187	0.94	1.00	1.16	1.37	1.47	18	82	0.95	1.00	1.14	1.35	1.48	18	105	0.93	1.00	1.17	1.38	1.48
24	182	0.89	0.95	1.10	1.32	1.45	24	80	0.92	0.96	1.08	1.30	1.46	24	102	0.88	0.95	1.12	1.33	1.45
Total	1032						Total	460						Total	572					
			**Smoothed centiles for SE (Shanon entropy) (All)**							**Smoothed centiles forSE (Shanon entropy) (Girls)**							**Smoothed centiles for SE (Shanon entropy) (Boys)**			
Months	n						Months	n						Months	n					
		3rd	10th	50th	90th	97th			3rd	10th	50th	90th	97th			3rd	10th	50th	90th	97th
0	260	2.32	2.45	2.76	3.07	3.20	0	117	2.31	2.45	2.76	3.05	3.16	0	143	2.33	2.46	2.76	3.07	3.20
6	205	2.49	2.61	2.89	3.17	3.28	6	95	2.49	2.61	2.88	3.15	3.27	6	110	2.50	2.62	2.90	3.17	3.29
12	198	2.59	2.71	2.97	3.24	3.36	12	86	2.60	2.70	2.95	3.23	3.35	12	112	2.59	2.71	2.98	3.25	3.36
18	187	2.62	2.74	3.01	3.30	3.42	18	82	2.64	2.74	2.99	3.28	3.43	18	105	2.60	2.73	3.02	3.30	3.41
24	182	2.57	2.70	3.00	3.32	3.47	24	80	2.62	2.73	2.99	3.32	3.48	24	102	2.54	2.68	3.01	3.32	3.45
Total	1032						Total	460						Total	572					
			**Smoothed centiles for CE (Conditional entropy) (All)**							**Smoothed centiles for CE (Conditional entropy) (Girls)**							**Smoothed centiles for CE (Conditional entropy) (Boys)**			
Months	n						Months	n						Months	n					
		3rd	10th	50th	90th	97th			3rd	10th	50th	90th	97th			3rd	10th	50th	90th	97th
0	260	0.53	0.57	0.66	0.77	0.83	0	117	0.52	0.56	0.66	0.76	0.80	0	143	0.53	0.57	0.66	0.78	0.84
6	205	0.61	0.65	0.73	0.83	0.87	6	95	0.61	0.64	0.73	0.82	0.86	6	110	0.62	0.65	0.74	0.83	0.88
12	198	0.66	0.70	0.78	0.87	0.91	12	86	0.66	0.69	0.77	0.86	0.90	12	112	0.67	0.70	0.78	0.87	0.91
18	187	0.67	0.71	0.80	0.89	0.93	18	82	0.68	0.71	0.79	0.88	0.92	18	105	0.67	0.71	0.80	0.89	0.93
24	182	0.65	0.69	0.79	0.89	0.93	24	80	0.66	0.70	0.78	0.88	0.93	24	102	0.64	0.69	0.79	0.89	0.93
Total	1032						Total	460						Total	572					
			**Smoothed centiles for CCE (Corrected conditional entropy) (All)**							**Smoothed centiles for CCE (Corrected conditional entropy) (Girls)**							**Smoothed centiles for CCE (Corrected conditional entropy) (Boys)**			
Months	n						Months	n						Months	n					
		3rd	10th	50th	90th	97th			3rd	10th	50th	90th	97th			3rd	10th	50th	90th	97th
0	260	0.56	0.60	0.71	0.84	0.90	0	117	0.55	0.60	0.71	0.82	0.88	0	143	0.57	0.61	0.71	0.84	0.91
6	205	0.64	0.68	0.78	0.89	0.94	6	95	0.64	0.68	0.77	0.88	0.93	6	110	0.65	0.69	0.78	0.89	0.95
12	198	0.69	0.73	0.82	0.92	0.98	12	86	0.69	0.72	0.81	0.92	0.97	12	112	0.69	0.73	0.83	0.93	0.98
18	187	0.70	0.74	0.84	0.94	0.99	18	82	0.70	0.74	0.83	0.94	0.99	18	105	0.70	0.74	0.84	0.95	1.00
24	182	0.67	0.72	0.83	0.95	1.00	24	80	0.68	0.72	0.82	0.94	1.00	24	102	0.66	0.71	0.83	0.95	1.01
Total	1032						Total	460						Total	572					
			**Smoothed centiles for NCCE (Normalized CCE) (All)**							**Smoothed centiles for NCCE (Normalized CCE) (Girls)**							**Smoothed centiles for NCCE (Normalized CCE) (Boys)**			
Months	n						Months	n						Months	n					
		3rd	10th	50th	90th	97th			3rd	10th	50th	90th	97th			3rd	10th	50th	90th	97th
0	260	0.43	0.46	0.53	0.62	0.67	0	117	0.42	0.45	0.52	0.61	0.64	0	143	0.44	0.47	0.53	0.62	0.68
6	205	0.50	0.52	0.59	0.67	0.70	6	95	0.49	0.52	0.58	0.65	0.69	6	110	0.50	0.53	0.60	0.67	0.71
12	198	0.53	0.56	0.63	0.70	0.72	12	86	0.53	0.56	0.62	0.69	0.71	12	112	0.53	0.57	0.64	0.70	0.73
18	187	0.54	0.58	0.64	0.71	0.73	18	82	0.55	0.57	0.63	0.70	0.73	18	105	0.54	0.58	0.65	0.72	0.74
24	182	0.53	0.56	0.63	0.70	0.73	24	80	0.54	0.56	0.63	0.70	0.73	24	102	0.53	0.57	0.64	0.71	0.73
Total	1032						Total	460						Total	572					
			**Smoothed centiles for rho (Entropy) (All)**							**Smoothed centiles for rho (Entropy) (Girls)**							**Smoothed centiles for rho (Entropy) (Boys)**			
Months	n						Months	n						Months	n					
		3rd	10th	50th	90th	97th			3rd	10th	50th	90th	97th			3rd	10th	50th	90th	97th
0	260	0.34	0.38	0.47	0.54	0.57	0	117	0.35	0.39	0.48	0.55	0.58	0	143	0.33	0.38	0.47	0.54	0.56
6	205	0.30	0.33	0.41	0.48	0.51	6	95	0.31	0.34	0.42	0.48	0.51	6	110	0.29	0.33	0.40	0.47	0.50
12	198	0.28	0.30	0.37	0.44	0.47	12	86	0.28	0.31	0.38	0.44	0.47	12	112	0.27	0.30	0.36	0.44	0.47
18	187	0.27	0.29	0.35	0.43	0.46	18	82	0.27	0.30	0.37	0.43	0.45	18	105	0.26	0.29	0.35	0.42	0.46
24	182	0.27	0.30	0.36	0.44	0.47	24	80	0.28	0.31	0.37	0.44	0.47	24	102	0.27	0.30	0.36	0.43	0.47
Total	1032						Total	460						Total	572					
			**Smoothed centiles for LZC (Lempel-Ziv Complexity) (All)**							**Smoothed centiles for LZC (Lempel-Ziv Complexity) (Girls)**							**Smoothed centiles for LZC (Lempel-Ziv Complexity) (Boys)**			
Months	n						Months	n						Months	n					
		3rd	10th	50th	90th	97th			3rd	10th	50th	90th	97th			3rd	10th	50th	90th	97th
0	260	0.95	0.97	1.00	1.03	1.03	0	117	0.95	0.97	1.00	1.02	1.03	0	143	0.95	0.97	1.01	1.03	1.03
6	205	0.90	0.92	0.96	1.00	1.01	6	95	0.89	0.91	0.96	1.00	1.01	6	110	0.90	0.92	0.96	1.00	1.00
12	198	0.84	0.87	0.93	0.97	0.99	12	86	0.84	0.87	0.93	0.97	0.99	12	112	0.85	0.88	0.93	0.97	0.98
18	187	0.80	0.84	0.91	0.96	0.97	18	82	0.78	0.83	0.91	0.96	0.97	18	105	0.81	0.85	0.91	0.96	0.97
24	182	0.76	0.81	0.89	0.96	0.97	24	80	0.74	0.79	0.89	0.95	0.96	24	102	0.78	0.82	0.90	0.96	0.97
Total	1032						Total	460						Total	572					

Entropy is a measure of the regularity and complexity of pattern of different length.

**Fig. 1:**
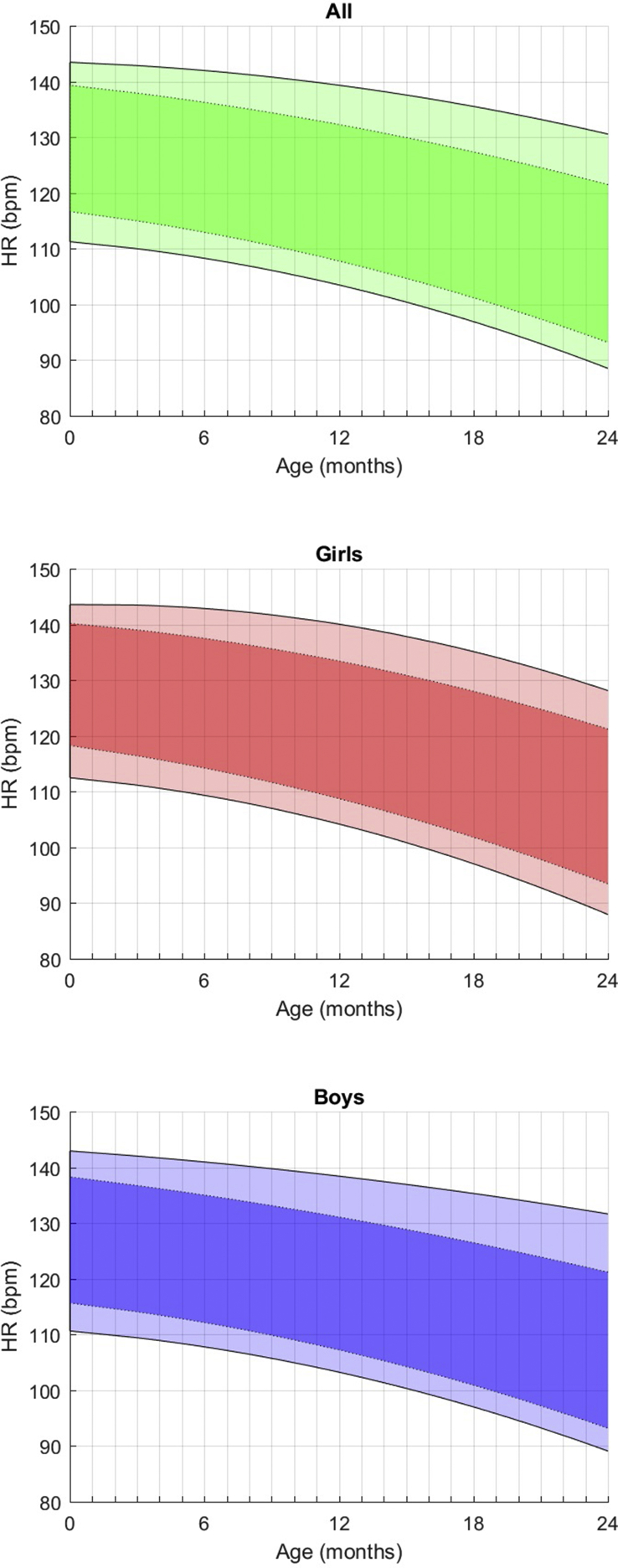
Fitted 3rd, 10th, 90th, and 97th smoothed centil curves (full lines) for mean heart rate according to age, for all children (upper panel) and separately for girls (middle panel) and boys (lower panel).

**Fig. 2:**
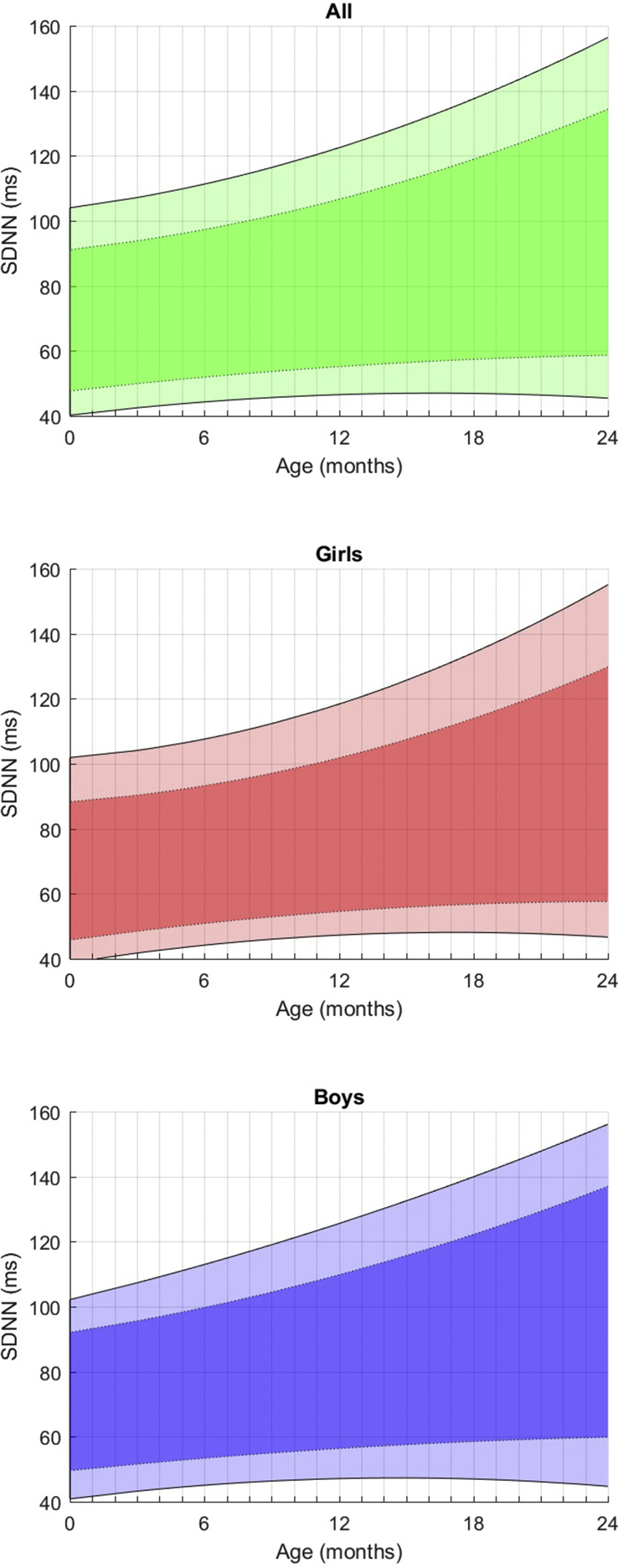
Fitted 3rd, 10th, 90th, and 97th smoothed centil curves for SDNN according to age, for all children (upper panel) and separately for girls (middle panel) and boys (lower panel).

**Fig. 3:**
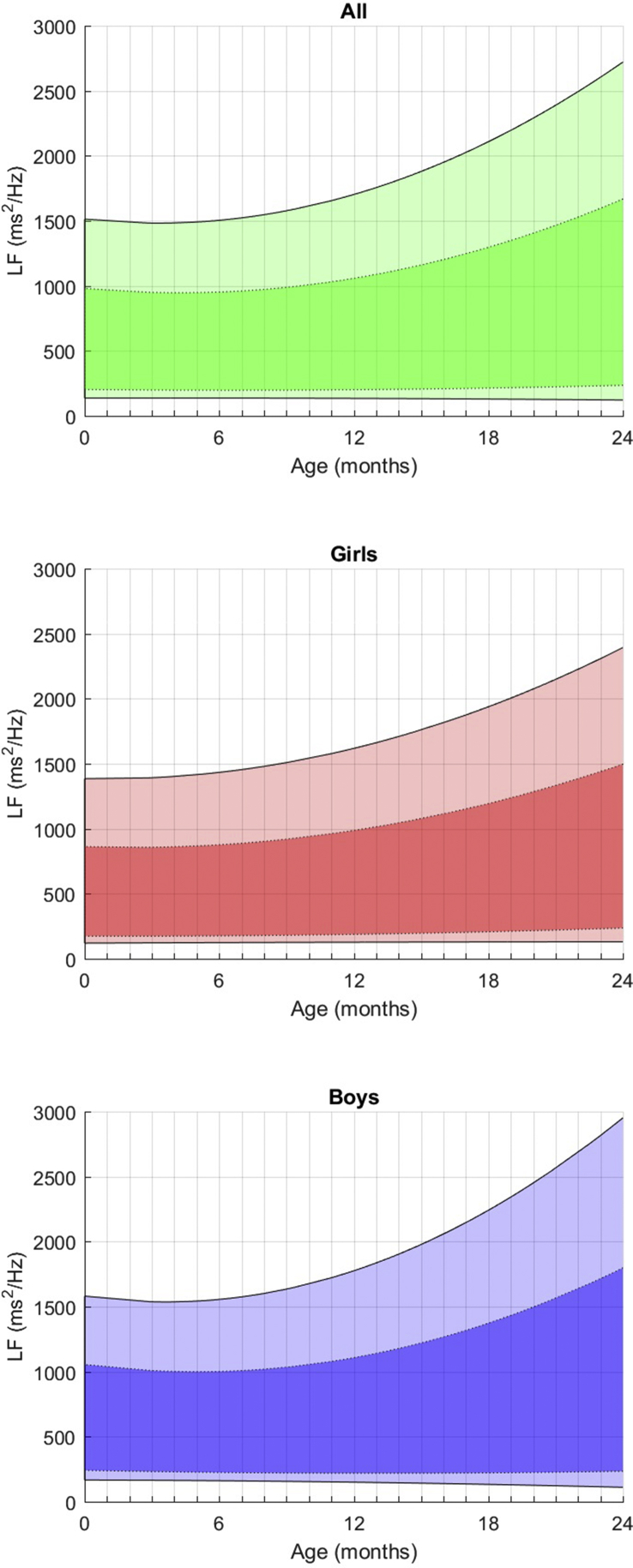
Fitted 3rd, 10th, 90th, and 97th smoothed centil curves for LF according to age, for all children (upper panel) and separately for girls (middle panel) and boys (lower panel).

**Fig. 4:**
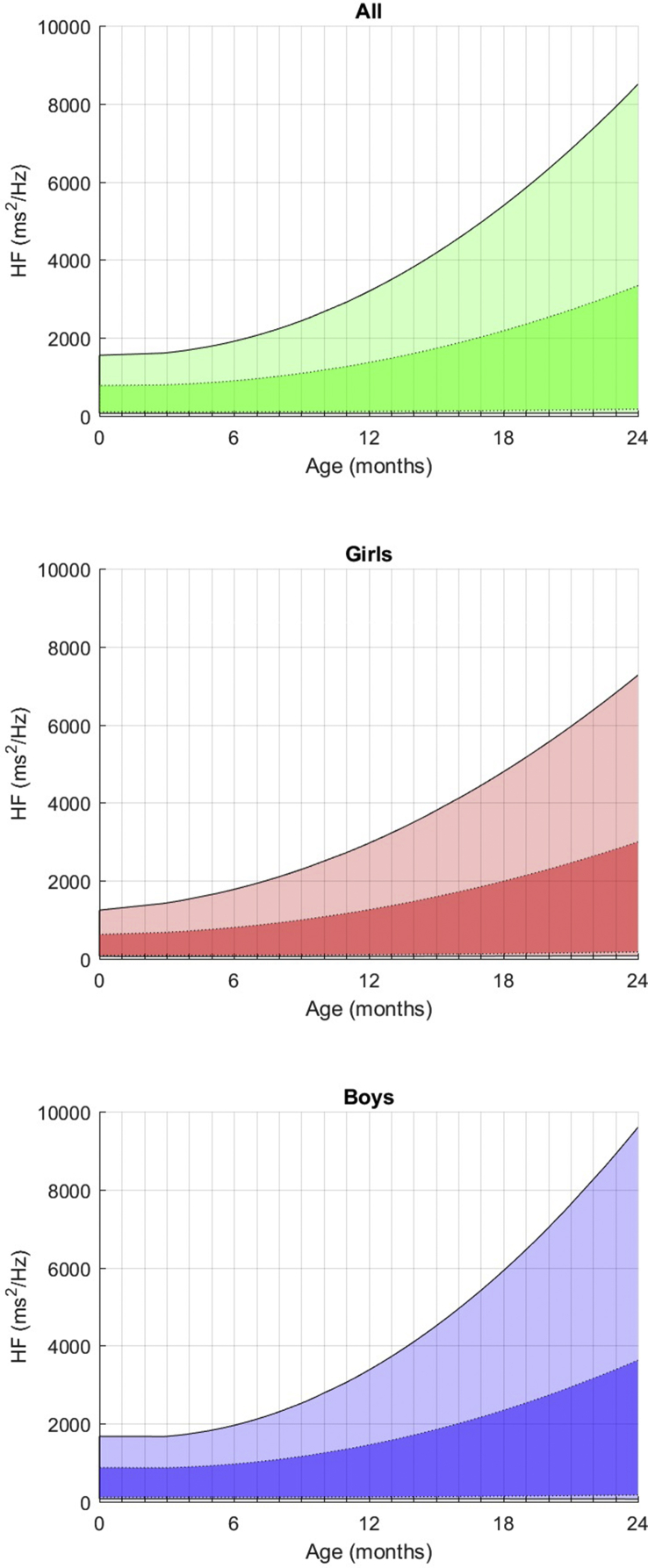
Fitted 3rd, 10th, 90th, and 97th smoothed centil curves for HF according to age, for all children (upper panel) and separately for girls (middle panel) and boys (lower panel).

More frequently used data are illustrated for HR, SDNN, LF and HF (Figs. 1, 2, 3, and 4).

### 3.1 HRV temporal and frequential approaches

**In the temporal-domain** (Table 1), the resting heart rate decreases regularly by 5 bpm on average every 6 months of age and is moderately lower, although not significantly, for boys than for girls, regardless of age.

In parallel, pNN20 increases regularly by 5% on average every 6 months of age, whereas pNN50 only increases by 1–2% over these same periods. These values are physiologically higher, although not significantly, for boys than for girls, regardless of age.

**For the geometrical indices** (Table 2), both the HRV triangular index and the TINN grow regularly from birth to age 2 regardless of gender.

**Fig. 5:**
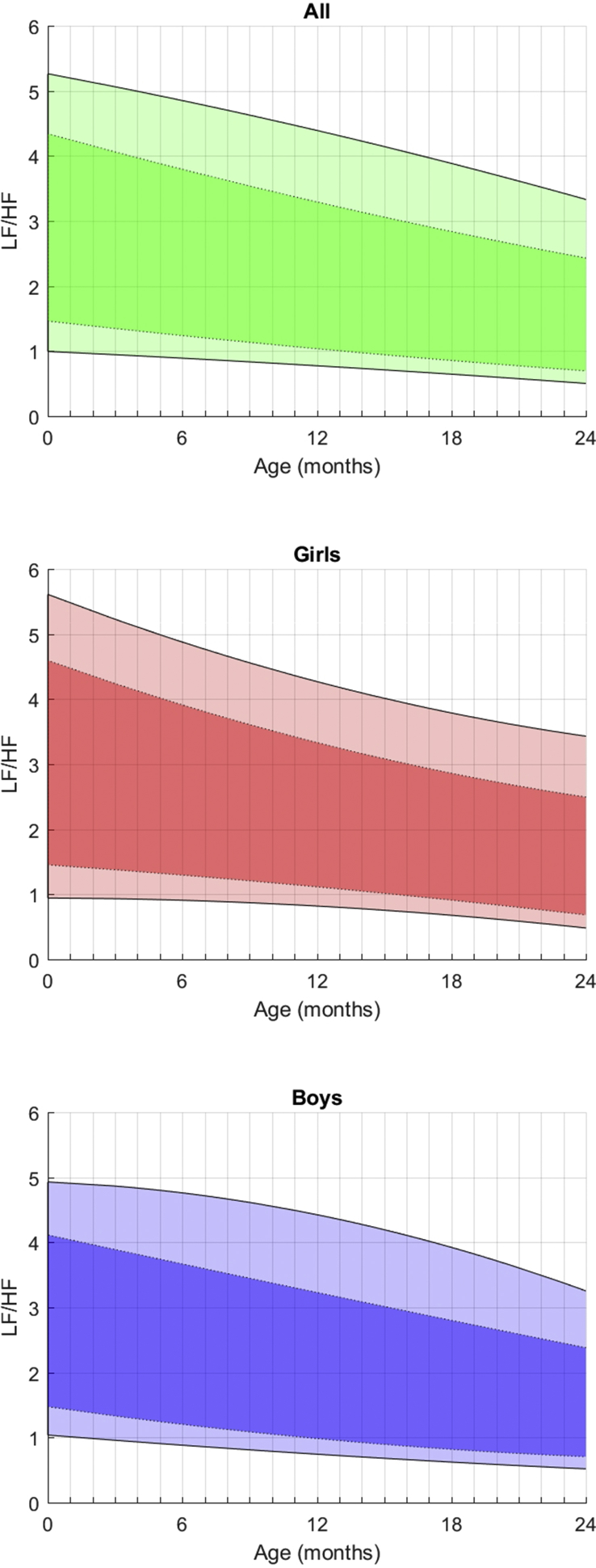
Fitted 3rd, 10th, 90th, and 97th smoothed centil curves for LF/HF ratio according to age, for all children (upper panel) and separately for girls (middle panel) and boys (lower panel).

**In the frequency domain** (Table 3), Ptot increases moderately in the first year while progressed exponentially afterwards, with a very large inter-individual variability. From birth, boys have a higher Ptot, which also progresses faster than girls (4081–5178 ms^2^/Hz vs 3341–4122 ms^2^/Hz). VLF values are very stable whatever the period considered and the gender. LF, which represents 12–13% of the entire frequency spectrum at this age, presents with a slow and steady increase of these values (435–648 ms^2^/Hz) between 0 and 2 years, with no noticeable peak and no major influence of gender, even if basal values are moderately higher for boys. The kinetics of maturation of the HF values is remarkable and very different from the LF values as, while it begins at low values, only 6–7% of the total spectrum, their progression is much faster and the values exceed the LF values at 2 years (14–15%), regardless of the genre. As a result, the LF/HF ratio decreases between birth and 2 years for both boys and girls (see Fig. 5).

### 3.2 HRV complexity by nonlinear approach

Poincaré plot SD1 increases regularly between 0 and 24 months (Table 4), being slightly higher in boys regardless the period. While SD1/SD2 ratio seems to increase with age, SD2 representing long-term variability has always values 4 to 6 times larger than SD1, as evidenced by the clear predominance of SD2nu and increases regularly with age.

Considering the Empirical Mode Decomposition (Table 5), we can observe that both low (pLF1, pLF2) and high (pHF1, and pHF2) frequencies increase from birth to age 2, as the ratio between low and high frequency indices (IMAI1 and IMAI2) decrease.

Indices of fractality (Table 6) decrease regularly with age, whatever the gender: the 2 exponents α1 and α2 of the detrended fluctuation analysis (DFA) used to quantify the degree of self-similarity of RR fluctuation [α1 for short-term fluctuation decreasing from 1.20 to 1, and α2 for long-term fluctuations decreasing from 1.05 to 0.9], H DFA decreasing 1.04 to 0.96 and Hurst exponent from 0.37 to 0.20.

Conversely, Higuchi and Katz algorithms proposed to determine the fractal dimension of heart rate variability signal increases respectively from 1.6 to 1.8 and from 1.4 to 1.6 over the same period.

The power-law slope (ß) influenced mainly by autonomic input to the heart quantifies the complexity of the RR interval. While the smaller the slope, the greater the loss of complexity, this index is however difficult to interpret in that population.

The “chaotic” exponent (Skewness, kurtosis and largest Lyapunov exponent) in our population increase respectively (0.39–0.57–5.82 to 6.23–0.23 to 0.3) meaning higher variations of the RR with age (Table 7).

If we consider the heart Deceleration and Acceleration Capacities indices used to estimate the vagal and sympathetic capacities, it is interesting to note that these two indices also progress with age in the same proportion, with a usual aspect in mirror.

What is remarkable in our population is the fact that all these entropy markers regularly increase by 20% on average between birth and 2 years (Table 8).

Another way to measure the rate of patterns recurrences in RR series is the Lempel-Ziv complexity. This last index decreases by 10% with age in our population.

## 4 Discussion

The main objective of the *Autonomic Baby Evaluation* cohort (*AuBE*) was to determine the physiological autonomic maturation profile from birth to 2 years in a healthy population of term neonates. This the first time such longitudinal survey was conducted in a large newborns healthy population.

In summary, during these two years of maturation, there is a large gain in global autonomic maturation giving progressively a new equilibrium privileging the parasympathetic activity over the sympathetic activity. This underlines a gain in fine-tuning autonomic modulations.

Thus the balance of the autonomic nervous system (ANS), essential for homeostasis and cardiorespiratory control, depends closely not only on states of wakefulness (awakening, quiet sleep, active sleep) [40, 41, 42, 43, 44] but also on postnatal age.

This study has provided a comprehensive analysis of HRV indices which may serve as reference data, are of interest in assessing global autonomic maturation. These markers have also gain some interest in pathological conditions as growth restricted and prematurity status [45], sepsis [23], inflammation [25], as well as in particular physiological settings as skin-to-skin [46], and stress or pain [47, 48].

The difference in values we measured from birth according to gender is notable. All HRV values in any field of analysis are slightly higher in boys. We do not have a rational explanation. This does not explain the higher risk of SIDS in premature male infants [49]. Conversely, it has been shown that girls presented significantly higher values than boys for SDNN and absolute high frequency (HF; p < 0.05) in the supine position, the most significant indices of the vagal activity [50]. We do not know when this occurs during childhood. For adults, females showed significantly lower mean RR interval and SDNN power spectral density but a significantly greater vagal activity [51].

There may be some limitations to our study. The first is related to the technical design of the study forcing us to analyze the data over 24 hours thus mixing waking and sleeping periods and day and night periods, which could change the basal values of HRV. In fact, we have dissociated sleep-wake data from polysomnography at birth and at 6 months of life, but it was no longer possible to obtain them on such a cohort, on an ambulatory basis and at an age when child is not compliant for physiological explorations. Nevertheless, the accumulation of 24-hour global data has the advantage of allowing measurements on a much larger number of RRs (more than 150,000 per day per child) and thus of attenuating the impact of brain activity stages on the results.

Mothers' sleep and mood could interfere with the child's sleep quality [52], while this was not taken into account in this results which thus includes such variations. There could be also unmeasured confounding factors as the impact of nicotine exposure during pregnancy which may target different organs of the fetus, particularly the lung and the central nervous system [53, 54], including learning disorders, hyperactivity and attentional deficits or moderate intellectual disabilities [55, 56, 57, 58, 59, 60]. Another limitation lies in the large standard deviations of normal values. For some indices, the data can vary from 1 to 20. It is therefore necessary to integrate this when used for a personalized follow-up. Each individual probably has his own autonomic resources predefined by his gender, and genetics as well as environmental factors. A human being his thus also its own witness able to improve his autonomic balance.

Using these tools may allow a complete non-invasive neurophysiological approach of the cardiorespiratory self-regulation. The innovative the longitudinal follow-up of healthy child allowed establishing normative data useful for the evaluation of an autonomic risk at a critical age of faintness and unexpected sudden death occurrence. Persistent dysautonomia in the neonatal period, as a biomarker of neuronal dysfunction, may warrant early and prolonged neurodevelopmental follow-up and perhaps corrective actions.

## 5 Conclusion

The physiological autonomic maturation profile from birth to 2 years in a healthy population of term neonates results in a fine autonomic maturation underlying increasingly a new equilibrium and benefitting the parasympathetic activity over the sympathetic activity.

## Declarations

### Author contribution statement

Hugues Patural: Conceived and designed the experiments; Wrote the paper.

Vincent Pichot: Analyzed and interpreted the data.

Sophie Flori, Antoine Giraud: Performed the experiments.

Patricia Franco, Patrick Pladys, Alain Beuchée: Contributed reagents, materials, analysis tools or data.

Jean-Claude Barthelemy, Frédéric Roche: Conceived and designed the experiments.

### Funding statement

The AuBE study is allowed through consecutive grants from the French Ministry of Health: PHRC interrégional, 2009 and AOL 2010.

### Competing interest statement

The authors declare no conflict of interest.

### Additional information

The clinical trial described in this paper was registered at ClinicalTrials.gov under the registration number NCT01583335.
